# Transcervical Fibroid Ablation (TFA): Update on Pregnancy Outcomes

**DOI:** 10.3390/jcm13102892

**Published:** 2024-05-14

**Authors:** Leslie Hansen-Lindner, Juliette Schmid-Lossberg, David Toub

**Affiliations:** 1 Atrium Health Women’s Care, Charlotte, NC 28204, USA; leslie.hansen-lindner@atriumhealth.org; 2 gynhealth AG, 8008 Zürich, Switzerland; schmid-lossberg@gyn-health.ch; 3 Medical Affairs, Gynesonics, Redwood City, CA 94063, USA

**Keywords:** uterine fibroid, leiomyoma, radiofrequency ablation, transcervical fibroid ablation, sonata system, pregnancy

## Abstract

**Background/Objectives:** Transcervical fibroid ablation (TFA) is an incisionless method to treat symptomatic uterine fibroids. While safety regarding future pregnancy remains to be established, TFA does not preclude the possibility of pregnancy, and a previous 36-patient case series of post-TFA pregnancies reported normal outcomes. That prior series did not include postmarket cases in the United States, as the Sonata^®^ System was initially cleared and used in Europe. This is a substantive update of known pregnancies with the Sonata System since June 2011, and includes pregnancies in Europe, Mexico, and the US. **Methods**: TFA was carried out under both clinical trial and postmarket use to treat symptomatic uterine fibroids. All post-TFA pregnancies reported by physicians with their patient’s consent were included. **Results**: 89 pregnancies and 55 deliveries have occurred among 72 women treated with the Sonata System. This includes 8 women who conceived more than once after TFA. Completed pregnancies (*n* = 62 women) include 19 vaginal deliveries, 35 Cesarean sections, 5 therapeutic abortions, 1 ectopic pregnancy, and 1 delivery by an unknown route. Ten pregnancies are ongoing. Mean birthweight was 3276.7 ± 587.3 g. Ten women experienced 18 first-trimester spontaneous abortions (SAbs), with 10 of the 18 SAbs (55.6%) occurring between two patients with a history of recurrent abortion. The SAb rate was 22.8%, inclusive of these two patients, and 10.1% if they were excluded as outliers. There were no instances of uterine rupture, placenta accreta spectrum, or stillbirth. **Conclusions**: This case series, the largest to date for any hyperthermic ablation modality, suggests that TFA with the Sonata System could be a feasible, safe treatment option regarding eventual pregnancy in women with symptomatic uterine fibroids.

## 1. Introduction

Uterine fibroids, or leiomyomata uteri, are highly prevalent, monoclonal benign tumors that can cause significant symptomatology in affected women. Symptoms include heavy menstrual bleeding, abdominopelvic pressure and discomfort, dyspareunia, and other symptoms. While there is a lack of definitive evidence that fibroids impair fertility regardless of location, myomectomy has been recommended as a consideration to improve pregnancy rates [1]. In addition to myomectomy, other alternatives to hysterectomy for women suffering from fibroids are available, including transcervical fibroid ablation (TFA). This incisionless, outpatient treatment option has been associated with procedural and postoperative safety, significant reductions in menstrual bleeding, prompt return to normal activity, durable and significant improvements in health-related quality of life (HRQOL), and symptom severity, with a cumulative reintervention rate of 8.2% through 36 months [2,3,4]. One study demonstrated continued benefits through more than 5 years, no surgical reinterventions through the first 3.5 years after TFA, and an 11.8% reintervention rate with a mean follow-up of 64 months [5].

The Sonata^®^ System (Gynesonics, Inc., Redwood City, CA, USA) has been cleared by the US Food & Drug Administration (FDA) and received the Conformite Europëenne (CE) Mark in the European Union. Under the FDA-cleared labeling for the Sonata System, there are no specific warnings or contraindications regarding a desire for future fertility. The FDA-cleared labeling does note that the safety regarding future pregnancy has not been established, and as a uterus-conserving hysterectomy alternative, the possibility of pregnancy is not precluded by treatment with Sonata. The Sonata System has been described elsewhere in detail [2,6,7].

The Sonata System integrates intrauterine ultrasound (IUUS) within the Sonata handpiece for imaging, providing real-time guidance during RF ablation. The IUUS image is noteworthy for several reasons, including the high resolution of intrauterine pathology. This is due to the IUUS probe’s location within the endometrial cavity that is closer to myometrium than a standard transvaginal ultrasound probe. There is also a graphical overlay (SMART Guide) that provides a targeting guide for treating gynecologists. This overlay is unique in that it depicts the ablation zone as well as a thermal safety border beyond which there are no material thermal tissue effects. This enables physicians to set the margins of ablation in real time, providing precision and control over where the desired ablation will take place and how large the ablation will be. The thermal safety border is unique among existing devices that use radiofrequency (RF) energy to ablate uterine fibroids and is a significant factor in the lack of reported thermal injuries to adjacent viscera to date. Multiple ablations may be performed within the same fibroid during a single treatment session, as when fibroids ≥ 5 cm are treated.

While there have been pregnancies reported after other hyperthermic ablative technologies such as focused ultrasound and percutaneous microwave ablation, case numbers have been relatively small, with the largest series consisting of 54 pregnancies after magnetic resonance-guided focused ultrasound (MRgFUS) [8,9]. There have also been pregnancies reported after laparoscopic fibroid ablation (LFA), with the largest published case series consisting of 30 pregnancies [10,11,12,13]. A small number of pregnancies (3 and 8, respectively), including one complicated by placenta accreta spectrum, have been included in two reports of single-needle, transvaginal RF ablation, which is not currently specifically indicated for fibroid treatment [14,15].

After initial single case reports of a normal spontaneous vaginal delivery (NSVD), an elective repeat Cesarean section (C/S), and a vaginal delivery status post assisted reproductive technology (ART) after TFA with the Sonata System, a case series detailing 36 pregnancies among 28 women was published in 2022 [16,17,18,19]. The authors of this case series noted that normal pregnancy outcomes were possible after TFA with the Sonata System. The initial experience with the Sonata System was confined to Europe and Mexico, outside of the 147-patient prospective multicenter SONATA clinical trial that included sites in the US, and all but one of the pregnancies in the 2022 case series occurred outside of the US. Since that case series, there has been a significant increase in the use of the FDA-cleared Sonata System within the US. This current case series thus includes the pregnancy experience after TFA in the US, as well as in the United Kingdom, Continental Europe, and Mexico.

## 2. Materials and Methods

This is a retrospective multicenter case series inclusive of all reported pregnancies after transcervical fibroid ablation at the time of this writing (March, 2024). TFA was performed on women either in clinical trials of the Sonata System (FAST-EU, SONATA, OPEN, SAGE) or in postmarket use (guided by the device’s Instructions for Use). Each clinical trial protocol is posted at ClinicalTrials.gov (FAST-EU, NCT01226290; SONATA, NCT02228174; OPEN, NCT02844920; SAGE, NCT03 118037), and each trial was supervised by their individual Ethics Committees and Institutional Review Boards. Physicians using Sonata included obstetrician/gynecologists in the US, Continental Europe, the UK and Mexico, and the physicians represented a variety of practice settings and management styles. Data regarding pregnancies were furnished by the clinical study sites (for patients enrolled in clinical trials) or individual treating physicians (for patients treated on a postmarket basis) after obtaining consent from the patients to share their anonymous data for this case series. These data were collected and maintained within a nonrelational database (Microsoft Excel, version 16; Microsoft, Redmond, WA, USA) that was used to perform descriptive analysis. Duplicative data, such as treated fibroid types and sizes in patients who delivered more than once after treatment with Sonata, were removed before analysis. Patient age was specified as the one at the time of conception; gravidity and parity were both exclusive of the current pregnancy.

## 3. Results

Eighty-nine pregnancies among 72 women treated with TFA for their symptomatic fibroids have been identified since the first such procedure in June 2011. Eight women have become pregnant more than once after TFA with the Sonata System; ten pregnancies are ongoing, and 11 women conceived as the result of ART. Twenty-six women who conceived were nulligravidae. There were no reported device- or procedure-related acute complications of TFA in this cohort of patients. Demographic information, including treated fibroid types using the International Federation of Gynecology and Obstetrics (FIGO) subclassification, is presented in Table 1 [20,21]. Most patients were treated on a postmarket basis. Of the clinical trial patients, one, two, six, and three women were enrolled in the FAST-EU, SONATA, SAGE, and OPEN clinical trials, respectively. Treated fibroid sizes ranged from 1.0 to 8.4 cm, and as many as six fibroids were ablated in individual patients. Regarding age ranges, 80.3% of patients were in their third decade of life (ages 31–40) with 61.8% in the range of 36–40 years of age.

While the data provided by the treating physicians do not include last menstrual period dating for most patients, the shortest documented intervals from TFA to conception include a woman in the FAST-EU trial who conceived approximately 3.5 months after TFA and underwent an uncomplicated repeat C/S at term, and another woman who conceived in a similar 3.1-month timeframe post-ablation (her pregnancy resulted in a first-trimester loss).

Sixty-two women have recorded pregnancy outcomes, including 55 deliveries. These outcomes include 19 vaginal deliveries, 35 Cesarean sections, 5 therapeutic abortions, 18 first-trimester spontaneous abortions (SAbs), 1 ectopic pregnancy, and 1 delivery by an unknown route. In the last case, the patient was ultimately lost to follow-up despite the treating physician’s best efforts, but he was able to establish that the patient had delivered at an outside institution, although the route of delivery was not known. Pregnancy outcomes are delineated in Table 2.

The mean birthweight was 3276.7 ± 587.3 g (median: 3355.0; range: 940–4500). Among the eight preterm neonates, the mean preterm gestational age was 33.7 ± 2.7 weeks (median: 35.0; range: 28–35.9). There were no instances of uterine rupture, placenta accreta spectrum, or stillbirth.

Most deliveries were by C/S, and the vast majority of Cesarean deliveries were primary sections. Cesarean sections were performed mainly for common obstetric indications such as failure to progress (FTP), breech presentation, fetal macrosomia, prior hysterotomy (C/S, myomectomy), nonreassuring fetal monitoring, or per patient request. Table 3 provides details for each C/S. A tabulation of the indications for C/S are shown in Table 4.

Ten women (14.1% of patients) experienced a total of 18 SAbs, all in the first trimester. In all, 10 of the 18 miscarriages (55.6%) occurred in two patients with an unfortunate history of recurrent abortion. As initially detailed in a prior report, one patient had a total of four first-trimester losses after treatment with Sonata, after having had a prior second-trimester miscarriage that was believed to have occurred secondary to a 3.9 cm type 2–5 fibroid [17]. She was evaluated for recurrent abortion and found to have antiphospholipid syndrome, a known risk factor for recurrent miscarriage [22]. That same patient subsequently had a full-term Cesarean delivery complicated by hemolysis with elevated liver enzymes and a low platelet count (HELLP syndrome). Since that initial report, the same patient has now had three further pregnancies: two first-trimester SAbs and one term vaginal delivery complicated again by pregnancy-induced hypertension. Thus, this one patient contributed to eight pregnancies after TFA: six first-trimester losses and two term deliveries (one C/S, one vaginal delivery). A second patient experienced four first-trimester losses after TFA, but prior to fibroid ablation, she had already suffered 16 miscarriages. The presumptive etiology for her large number of recurrent miscarriages was not established.

## 4. Discussion

Transcervical fibroid ablation using the Sonata System is an incisionless treatment option that has demonstrated both safety and durable effectiveness. It may be considered a potential treatment option for any woman with symptomatic uterine fibroids, and has similar effectiveness to LFA but through a transcervical route [6,23]. The presence of the SMART Guide, with both a graphical Ablation Zone and a validated Thermal Safety Border, enables treating physicians to target fibroid tissue under their own control and with precision. Thus, gynecologists may selectively target and treat fibroids and avoid adjacent endometrium and/or myometrium as desired for their patients, potentially minimizing effects of coagulative necrosis on surrounding tissue [24].

The cumulative experience with TFA, which has been used to treat more than 8000 women globally since 2011, has been reassuring with respect to future pregnancy. The Sonata System has demonstrated rates of postpartum hemorrhage, preterm delivery, low five-minute Apgar score, and low birth weight that are less than or similar to those of the general population.

Postpartum hemorrhage (PPH) is defined as an estimated blood loss of >1 L along with clinical signs/symptoms of hypovolemia within 24 h postpartum, regardless of delivery route [25]. Rates of PPH have increased and been reported as high as 4.3% based on an analysis of 76.7 million postpartum admissions [26]. The rate of PPH seen after TFA (7.3%) may reflect both the variability inherent in a smaller cohort, subjective estimates of blood loss without corresponding stigmata of true hypovolemia, as well as the presence of new and/or untreated fibroids, which are associated with increased puerperal blood loss [27]. The preterm delivery rate (14.5%) is similar to the global prematurity rate of around 11%, although rates vary widely among ethnicities and various countries [28]. The rate of low birthweight (3.8%) compares favorably against the estimated rate of 7% for North America, Europe, Australia, and New Zealand in 2015 [29].

The overall SAb rate after TFA was 22.8%, which is lower than what has recently been reported for laparoscopic fibroid ablation (40.9%) in the midterm analysis of the ULTRA study, although that was based on only 22 pregnancies in the LFA arm and was similar to the 42.9% SAb rate among the 21 pregnancies in the myomectomy arm [10]. Two of the ten patients in our case series who experienced pregnancy losses after TFA, all in the first trimester, were outliers due to their unfortunate significant history and, therefore, an increased risk of recurrent miscarriage. Nonetheless, one of these two women did experience two term deliveries after treatment with the Sonata System. Without the pregnancy losses from these two patients, the rate of miscarriage after TFA would be 10.1% (8 of 79 completed pregnancies), which is below the 13.3% rate reported for the largest individual case series of 30 pregnancies to date after LFA. It should also be noted that the general background rate for spontaneous abortion has been provided as 43%, and it increases with increasing age, with an estimated 8.7% rate by the age of 22 years to 84.1% by 48 years of age and higher [30,31]. The cumulative overall risk of pregnancy loss based on gestational age from weeks 5 to 20 has been estimated to range from 11 to 22% [32].

There were no cases of uterine rupture, stillbirth, or placenta accreta spectrum. That said, the number of deliveries, especially per vaginam, is limited, and the true incidence of uterine rupture and other complications during or prior to labor cannot be estimated after treatment with the Sonata System. Uterine ruptures, including associated neonatal mortality, have been infrequently reported for other hyperthermic ablation modalities such as focused ultrasound (FUS) and LFA [10,33,34]. While uterine rupture after either C/S, myomectomy, or hyperthermic fibroid ablation fortunately remains rare, gynecologists who perform TFA must be cognizant of the possibilities of uterine rupture and other potential pregnancy-related complications when selecting patients for the procedure. It remains to be determined whether vaginal or abdominal delivery is most appropriate after TFA.

The optimal timing of conception post-ablation is unknown and remains to be established, whether for natural conception or ART. While the earliest documented pregnancies involved conceptions just over 3 months after TFA, it is not currently possible to establish the optimal waiting period for natural or ART-based conception. It is conceivable that the coagulative necrosis process, which is inflammatory in nature, could inhibit blastocyst implantation or affect placentation [24].

There were 35 Cesarean sections, representing 63.6% of deliveries, of which 29 represented primary C/S procedures. The indications for C/S were mainly for patient preference, as well as common obstetrical indications such as breech presentation, cephalopelvic disproportion/failure to progress, and nonreassuring antenatal testing. Nonetheless, the C/S rate is higher than the 54.5% rate reported by Allen and colleagues (*n* = 22 pregnancies) and the 50% rate noted by Berman and colleagues (*n* = 30 pregnancies) in their reports of pregnancies after LFA [10,13]. The high percentage of Cesarean sections for patient preference, mostly for primary C/S, may reflect a wider acceptance of patient choice C/S in Europe where all such cases occurred, compared with the US where the prevalence of C/S on patient request is only 2.6% [35]. Habiba and colleagues noted a relatively high acceptance of patient choice C/S in Germany and the UK [36]. Indeed, treatment sites in Germany along with Switzerland contributed a significant number of patients to this case series, including some of the elective primary Cesarean sections.

Strengths of this case series include the high percentage of patients treated on a postmarket basis as well as its global nature and variation in practice setting, which may be more representative than controlled clinical trials in terms of real-world outcomes with pregnancy after TFA. The most frequently treated fibroids were type 2–5 and type 3 fibroids, both of which either indent or abut the endometrial cavity and may negatively affect pregnancy, although the evidence is not yet definitive [1,37,38]. At the same time, the presence of postmarket data regarding pregnancy outcomes suggests that it is highly likely that there are outstanding data for other pregnancies that are not captured in this case series. This could include both positive and negative outcomes. Most deliveries were by C/S, limiting the experience with vaginal delivery to 19 instances, which is not sufficient to guide whether surgical or vaginal delivery is preferred for pregnancies after TFA. While it is encouraging that no instances of uterine rupture or placenta accreta spectrum were observed, the total number of deliveries (55) are insufficient to know if either or both complications will remain unseen or rare as the pregnancy experience after TFA grows. Thus, this is a limited dataset, and this should be considered by physicians and their patients when contemplating TFA as a treatment option.

Nonetheless, this uniquely large case series of post-TFA pregnancies provides information for gynecologists that may be useful when weighing treatment options with their patients suffering from uterine fibroids who desire pregnancy or have not excluded future fertility. Women deserve choices in both their fibroid treatment and reproductive options; TFA with the Sonata System provides an incisionless, durable, and safe treatment option that has now been associated with nearly 100 pregnancies to date with generally positive outcomes.

## 5. Conclusions

This case series, the largest to date for any hyperthermic ablation modality, suggests that conception and normal, term pregnancy outcomes can be achieved after TFA with the Sonata System. There were no instances of uterine rupture, stillbirth, or placenta accreta spectrum.

## Figures and Tables

**Table 1:** Patient and ablated fibroid characteristics.

Characteristic	Result	Percentage
Unique patients (*n*)	72	
Age (mean ± SD) at conception (years)	37.2 ± 4.8	
Gravidity (mean ± SD) ^1^	2.5 ± 4.2	
Parity (mean ± SD) ^1^	0.5 ± 0.9	
Nulligravidae (*n*)	26	
Clinical trial patients (*n*)	12	16.7%
Patients treated postmarket (*n*)	60	83.3%
Total no. of fibroids ablated (*n*)	101	
Ablated fibroid diameter (mean ± SD; cm)	3.3 ± 1.6	
No. of fibroids ablated/patient (mean ± SD)	1.7 ± 1.3	
Ablated type 1 fibroids (*n*)	4	4.0%
Ablated type 2 fibroids (*n*)	14	13.9%
Ablated type 3 fibroids (*n*)	21	20.8%
Ablated type 4 fibroids (*n*)	15	14.9%
Ablated type 5 fibroids (*n*)	14	13.9%
Ablated type 6 fibroids (*n*)	2	2.0%
Ablated type 2–5 or type 3–5 fibroids (*n*)	31	30.7%

TFA = transcervical fibroid ablation; SD = standard deviation; ^1^ gravidity and parity are all considered prior to each post-TFA pregnancy.

**Table 2:** Pregnancies and outcomes.

Result	*n*	Percentage
Total no. of pregnancies	89	
Completed (*n* = 62 women)	79	88.8% ^1^
Ongoing pregnancies	10	11.2% ^1^
Lost to follow-up (but delivered, unknown route)	1	1.1% ^1^
Patients with >1 pregnancy s/p TFA	8	9.0% ^1^
Patients who conceived via ART	11	12.4% ^1^
Deliveries (≥20 weeks)	55	69.6% ^2^
Clinical trial deliveries	8	14.5% ^3^
Postmarket deliveries	47	85.5% ^3^
Vaginal deliveries	19	34.5% ^3^
Cesarean sections	35	63.6% ^3^
Primary C/S	29	82.9%
Repeat C/S	6	17.1%
Unknown route	1	1.3% ^2^
Abortions	23	29.1% ^2^
TAb	5	6.3% ^2^
1st Trimester SAb (10 women)	18	22.8% ^2^
Ectopic pregnancy	1	1.3% ^2^
Stillbirth	0	0.0% ^3^
PTD (<37 weeks’ gestational age)	8	14.5% ^3^
Uterine rupture	0	0.0% ^3^
Postpartum hemorrhage	4	7.3% ^3^
Placenta accreta spectrum	0	0.0% ^3^
Five-minute Apgar score < 7	1	1.3% ^3^
Birthweight < 2500 g	3	3.8% ^3^

C/S = Cesarean section; TFA = transcervical fibroid ablation; TAb = therapeutic (elective) abortion; SAb = spontaneous abortion; ART = assisted reproductive technology; PTD = preterm delivery; ^1^ Denominator = total pregnancies (*n* = 89); ^2^ Denominator = completed pregnancies (*n* = 79); and ^3^ Denominator = deliveries (*n* = 55).

**Table 3:** Details of Cesarean sections (*n* = 35 women; 35 events) after treatment with TFA.

Index	Type of C/S	Gestational Age (Weeks)	Indication (s)
1	Primary	38 6/7	Patient preference
2	Primary	38 2/7	Fetal macrosomia (4500 g)
3	Primary	40 5/7	Nonreassuring fetal tracing, failed TOL, HELLP syndrome
4	Primary	41 3/7	FTP
5	Primary	38 5/7	Fetal macrosomia (4040 g)
6	Primary	39 1/7	Breech presentation
7	Primary	39 6/7	Nonreassuring fetal tracing, PROM, MSAF
8	Primary	39 1/7	Patient preference
9	Repeat	38 1/7	Prior C/S × 2
10	Repeat	38 2/7	Fetal macrosomia (4005 g)
11	Repeat	38 1/7	Patient preference
12	Primary	39 4/7	FTP, nonreassuring fetal tracing
13	Primary	38 2/7	Patient preference
14	Repeat	39 5/7	Patient preference
15	Primary	40	Pelvic outlet obstruction due to fibroid/FTP
16	Primary	35	FTP due to new 7 cm type 6 myoma
17	Primary	41 0/7	FTP
18	Primary	39 5/7	Patient preference
19	Primary	37	Oligohydramnios, transverse lie, PIH
20	Primary	35 2/7	PTL, multiple fibroids
21	Primary	32 1/7	PROM
22	Primary	40 1/7	FTP/maternal exhaustion
23	Primary	38 4/7	Patient preference after ART (ICSI/ET)
24	Primary	39	Patient preference
25	Primary	28	PROM/PTL, nonreassuring fetal tracing
26	Primary	37 3/7	S/P multiple prior myomectomies (two hysteroscopic, one laparoscopic) and TFA
27	Primary	37 weeks	LLP
28	Primary	39	Patient preference
29	Primary	39	S/P prior myomectomy
30	Primary	39	Patient preference
31	Repeat	35 3/7	h/o previa accreta and prior myomectomy
32	Primary	37	h/o myomectomy
33	Primary	35 1/7	Polyhydramnios, breech presentation, abnormal fetal MCA velocimetry
34	Repeat	37 4/7	h/o prior C/S
35	Primary	33	PROM, anhydramnios, patient preference

C/S = Cesarean section; h/o = history of; TOL = trial of labor; HELLP = hemolysis with elevated liver enzymes and a low platelet count; FTP = failure to progress; LLP = low-lying placenta; PTL = preterm labor; PROM = premature rupture of membranes; MSAF = meconium-stained amniotic fluid; PIH = pregnancy-induced hypertension; S/P = status post; TFA = transcervical fibroid ablation; and MCA = middle cerebral artery.

**Table 4:** Most frequent indications for a Cesarean section.

Indication	*n*	Percentage of C/S *
Patient preference	11	31.4%
FTP	6	17.1%
Prior hysterotomy	6	17.1%
Nonreassuring antenatal testing	5	14.3%
PROM	4	11.4%
Fetal macrosomia	3	8.6%
Breech presentation	2	5.7%

FTP = failure to progress; PROM = premature rupture of membranes; * Percentages sum to more than 100% due to multiple indications in individual patients.

## Data Availability

The obstetrical clinical data presented in this article are not readily available to maintain privacy of the treating physicians and their patients.

## References

[1] ASRM (2017). Removal of myomas in asymptomatic patients to improve fertility and/or reduce miscarriage rate: A guideline. Fertil. Steril..

[2] Chudnoff S., Guido R., Roy K., Levine D., Mihalov L., Garza-Leal J.G. (2019). Ultrasound-Guided Transcervical Ablation of Uterine Leiomyomas. Obstet. Gynecol..

[3] Lukes A., Green M.A. (2020). Three-Year Results of the SONATA Pivotal Trial of Transcervical Fibroid Ablation for Symptomatic Uterine Myomata. J. Gynecol. Surg..

[4] Roy K., Robinson J.K. (2022). Durable Improvement in Generic and Fibroid-Specific Quality of Life in Women Treated with Transcervical Fibroid Ablation with the Sonata System After Three Years. J. Gynecol. Surg..

[5] Garza-Leal J.G. (2019). Long-Term Clinical Outcomes of Transcervical Radiofrequency Ablation of Uterine Fibroids: The VITALITY Study. J. Gynecol. Surg..

[6] Lindner L.H., Roy K., Toub D.B. (2022). Transcervical Fibroid Ablation (TFA) with the Sonata System: Updated Review of a New Paradigm for Myoma Treatment. Curr. Obstet. Gynecol. Rep..

[7] Toub D.B. (2017). A New Paradigm for Uterine Fibroid Treatment: Transcervical, Intrauterine Sonography-Guided Radiofrequency Ablation of Uterine Fibroids with the Sonata System. Curr. Obstet. Gynecol. Rep..

[8] Rabinovici J., David M., Fukunishi H., Morita Y., Gostout B.S., Stewart E.A. (2010). Pregnancy outcome after magnetic resonance-guided focused ultrasound surgery (MRgFUS) for conservative treatment of uterine fibroids. Fertil. Steril..

[9] Bing-Song Z., Jing Z., Zhi-Yu H., Chang-Tao X., Rui-Fang X., Xiu-Mei L., Hui L. (2016). Unplanned pregnancy after ultrasound-guided percutaneous microwave ablation of uterine fibroids: A follow-up study. Sci. Rep..

[10] Allen A., Schembri M.B., Parvataneni R., Waetjen L.E., Varon S., Salamat-Saberi N., Tassone S., Williams N., Kho K.A., Jacoby V.L.M. (2024). Pregnancy Outcomes After Laparoscopic Radiofrequency Ablation of Uterine Leiomyomas Compared With Myomectomy. Obstet. Gynecol..

[11] Berman J.M., Puscheck E.E., Diamond M.P. (2012). Full-term vaginal live birth after laparoscopic radiofrequency ablation of a large, symptomatic intramural fibroid: A case report. J. Reprod. Med..

[12] Berman J.M., Bolnick J.M., Pemueller R.R., Leal J.G.G. (2015). Reproductive Outcomes in Women Following Radiofrequency Volumetric Thermal Ablation of Symptomatic Fibroids. A Retrospective Case Series Analysis. J. Reprod. Med..

[13] Berman J.M., Shashoua A., Olson C., Brucker S., Thiel J.A., Bhagavath B. (2020). Case Series of Reproductive Outcomes after Laparoscopic Radiofrequency Ablation of Symptomatic Myomas. J. Minim. Invasive Gynecol..

[14] Kim C.-H., Lee H.-A., Kim S.-H., Chae H.-D., Kang B.-M. (2011). Transvaginal ultrasound-guided radiofrequency myolysis for uterine myomas. Hum. Reprod..

[15] Santalla-Hernández A., Manzanares S., Naveiro-Fuentes M., López-Criado M.S., Fernández-Parra J. (2023). Obstetric outcome after ultrasound guided transvaginal radiofrequency ablation of uterine myomas. Obstet. Gynecol. Int. J..

[16] Bends R., Toub D.B., Römer T. (2018). Normal spontaneous vaginal delivery after transcervical radiofrequency ablation of uterine fibroids: A case report. Int. J. Women Health.

[17] Christoffel L., Bends R., Toub D., Schiermeier S., Pschadka G., Engelhardt M., Quinn S., Hartmann M., Habiba M., Felberbaum R. (2022). Pregnancy Outcomes After Transcervical Radiofrequency Ablation of Uterine Fibroids with the Sonata System. J. Gynecol. Surg..

[18] Garza-Leal J.G., León I.H., Toub D. (2014). Pregnancy after transcervical radiofrequency ablation guided by intrauterine sonography: Case report. Gynecol. Surg. Surgery.

[19] Pschadka G., Engelhardt M., Niehoff C., Toub D. (2019). Term Delivery in an Infertile Patient after Transcervical Radiofrequency Fibroid Ablation and Assisted Reproductive Technology. J. Gynecol. Surg..

[20] Munro M.G., Critchley H.O., Broder M.S., Fraser I.S., for the FIGO Working Group on Menstrual Disorders (2011). FIGO classification system (PALM-COEIN) for causes of abnormal uterine bleeding in nongravid women of reproductive age. Int. J. Gynaecol. Obstet..

[21] Munro M.G., Critchley H.O., Fraser I.S., The FIGO Menstrual Disorders Committee (2018). The two FIGO systems for normal and abnormal uterine bleeding symptoms and classification of causes of abnormal uterine bleeding in the reproductive years: 2018 revisions. Int. J. Gynaecol. Obstet..

[22] Sammaritano L.R. (2020). Antiphospholipid syndrome. Best Pract. Res. Clin. Rheumatol..

[23] ACOG (2021). Management of Symptomatic Uterine Leiomyomas: ACOG Practice Bulletin, Number 228. Obstet. Gynecol..

[24] Keltz J., Levie M., Chudnoff S. (2017). Pregnancy Outcomes After Direct Uterine Myoma Thermal Ablation: Review of the Literature. J. Minim. Invasive Gynecol..

[25] ACOG (2006). Postpartum Hemorrhage: ACOG Practice Bulletin, Number 76. Obstet. Gynecol..

[26] Corbetta-Rastelli C.M., Friedman A.M., Sobhani N.C., Arditi B.M., Goffman D., Wen T. (2023). Postpartum Hemorrhage Trends and Outcomes in the United States, 2000–2019. Obstet. Gynecol..

[27] Choudhary A., Inamdar S.A., Sharma U. (2023). Pregnancy With Uterine Fibroids: Obstetric Outcome at a Tertiary Care Hospital of Central India. Cureus.

[28] Walani S.R. (2020). Global burden of preterm birth. Int. J. Of. Gynecol. Obstet..

[29] Blencowe H., Krasevec J., de Onis M., E Black R., An X., A Stevens G., Borghi E., Hayashi C., Estevez D., Cegolon L. (2019). National, regional, and worldwide estimates of low birthweight in 2015, with trends from 2000: A systematic analysis. Lancet Glob. Health.

[30] Cohain J.S., Buxbaum R.E., Mankuta D. (2017). Spontaneous first trimester miscarriage rates per woman among parous women with 1 or more pregnancies of 24 weeks or more. BMC Pregnancy Childbirth.

[31] Nybo Andersen A.M., Wohlfahrt J., Christens P., Olsen J., Melbye M. (2000). Maternal age and fetal loss: Population based register linkage study. BMJ.

[32] Ammon Avalos L., Galindo C., Li D. (2012). A systematic review to calculate background miscarriage rates using life table analysis. Birth Defects Res. Part A Clin. Mol. Teratol..

[33] Lai T.H.T., Seto M.T.Y., Cheung V.Y.T. (2022). Intrapartum uterine rupture following ultrasound-guided high-intensity focused ultrasound ablation of uterine fibroid and adenomyosis. Ultrasound Obs. Gynecol..

[34] Li X.-W., Liang M.-Y., Wang J.-L., Wang D.-P. (2015). Spontaneous Uterine Rupture during Late Pregnancy after High-intensity Focused Ultrasound. Chin. Med. J..

[35] ACOG (2019). Cesarean Delivery on Maternal Request. ACOG Committee Opinion No. 761. American College of Obstetricians and Gynecologists. Obstet. Gynecol..

[36] Habiba M., Kaminski M., Da Frè M., Marsal K., Bleker O., Librero J., Grandjean H., Gratia P., Guaschino S., Heyl W. (2006). Caesarean section on request: A comparison of obstetricians’ attitudes in eight European countries. BJOG Int. J. Obstet. Gynaecol..

[37] Bai X., Lin Y., Chen Y., Ma C. (2020). The impact of FIGO type 3 fibroids on in-vitro fertilization outcomes: A nested retrospective case-control study. Eur. J. Obstet. Gynecol. Reprod. Biol..

[38] Brady P.C., Stanic A.K., Styer A.K. (2013). Uterine fibroids and subfertility: An update on the role of myomectomy. Curr. Opin. Obstet. Gynecol..

